# Encapsulation of Curcumin and Gemcitabine: Cytotoxic Effect and Mechanisms of Death in Lung Cancer

**DOI:** 10.1155/adpp/8816364

**Published:** 2025-08-13

**Authors:** Oscar Alberto Alvarez-Quezada, Norma Cesilia Arellano-Rodríguez, Mara Valeria Rodríguez-Rodríguez, Moisés Armides Franco-Molina, Diana Ginette Zarate-Triviño, Diana Elisa Zamora-Ávila, Claudia Lucía Vargas-Requena, Pablo Zapata-Benavides, María Cristina Rodríguez-Padilla

**Affiliations:** ^1^Departamento de Microbiología e Inmunología, Facultad de Ciencias Biológicas, Universidad Autónoma de Nuevo León (UANL), San Nicolás de los Garza 66450, Nuevo León, Mexico; ^2^Departamento de Ciencias Químico-Biológicas, Instituto de Ciencias Biomédicas, Universidad Autónoma de Ciudad Juárez (UACJ), Ciudad Juárez 32310, Chihuahua, Mexico; ^3^Departamento de Genética, Facultad de Medicina Veterinaria, Universidad Autónoma de Nuevo León (UANL), General Escobedo 66050, Nuevo León, Mexico

**Keywords:** curcumin, cytotoxic effect, gemcitabine, lung cancer, nanoparticles

## Abstract

Lung cancer is the second most common type of cancer and the leading cause of cancer-related deaths worldwide. Some chemotherapeutic agents, such as curcumin and gemcitabine, have low bioavailability due to their hydrophobicity or the need for specialized transporters. This limits their cytotoxic potential against tumor cells but can be addressed through nanoencapsulation. This study evaluated the effects of nanometric encapsulation of curcumin and gemcitabine in chitosan, a biocompatible polymer, on the A549 lung cancer cell line and B16F10 murine melanoma cells. The chemical properties of the synthesized nanoparticles were characterized using UV-vis spectroscopy, Fourier transform infrared (FTIR) spectroscopy, and scanning electron microscopy (SEM). The nanoparticles ranged in size from 180 to 197 nm, with a positive surface charge between 11.8 and 16.3 mV. Cytotoxicity assays were conducted on the A549 and B16F10 cell lines, along with morphological analyses of apoptosis and flow cytometry to assess cell death mechanisms. Compared to the free drugs, the nanometric encapsulation of curcumin and gemcitabine did not always enhance the cytotoxic effects, but it did induce pronounced apoptosis in the lung cancer cells. These findings suggest that this approach could optimize drug delivery, reduce the required doses, and minimize side effects, thereby improving the overall efficacy of lung cancer treatment.

## 1. Introduction

Lung cancer is a high-priority health concern affecting millions of people worldwide. It is the second most common type of cancer and the leading cause of cancer-related deaths. Each year, 1.6 million new cases are diagnosed and one million deaths are recorded [1]. Most lung cancer patients face a poor prognosis, with a 5-year survival rate of less than 15% following diagnosis [2]. In cases of metastatic lung cancer, survival drops to 4%. Smoking, both active and passive, remains the primary risk factor for developing this disease [3, 4]. The introduction of relatively new drugs has raised expectations for lung cancer treatment. Gemcitabine, a first-line chemotherapeutic agent for lung cancer [5, 6], has shown limitations in its absorption when tumor cells express low levels of nucleoside transporters such as human equilibrative nucleoside transporter 1 (hENT1), reducing its efficacy [7–10]. As a result, combining gemcitabine with other agents that enhance its effect, such as cisplatin, which has synergistic effects, is a promising approach [11, 12].

Curcumin is a natural compound with antitumor effects on various cancers, including lung cancer [13, 14]. Its mechanism of action involves altering multiple cell signaling pathways that regulate cell proliferation, survival, and death [15–17]. Due to this, curcumin has been used as an adjuvant in chemotherapy, increasing tumor cell sensitivity to drugs [18–20]. However, its polarity limits its water solubility and compromises its bioavailability. Therefore, it is crucial to develop strategies that improve the absorption of active ingredients. Encapsulating polymers like chitosan enhance drug protection, increase circulating half-life, and improve absorption [21–24]. Additionally, they promote better biodistribution of hydrophobic compounds [25, 26], enhancing the therapeutic effects of active ingredients while reducing potential side effects [27–29]. This study aimed to evaluate the effects of curcumin and gemcitabine nanoencapsulation on the cell death mechanisms of A549 lung cancer cell lines and B16F10 murine melanoma. The latter serves as a model for inducing metastatic pulmonary melanoma in C57BL/6 mice. A novel method for the delivery of curcumin and gemcitabine is proposed, with the potential to enhance their therapeutic effects against pulmonary neoplasms.

## 2. Materials and Methods

### 2.1. Development of Nanoparticles

The preparation of chitosan nanoparticles (Np-Q) was carried out using a chitosan solution in 0.1 M acetic acid at pH 5, with a concentration of 1 mg/mL, which was maintained overnight. The solution was filtered through a 0.45 μm membrane with constant stirring, and 50 μL of TPP solution (2 mg/mL) was added every 2 s. Chitosan and TPP were used at a 5:1 ratio. Subsequently, the mixture was centrifuged at 3000 rpm for 15 min, after which the supernatant was recovered for further analysis. For curcumin particles, 400 μL of curcumin in methanol at a concentration of 2.5 mg/mL was added to the chitosan solution (Np-Cur), while for gemcitabine particles (Np-Gem), 1.25 μL at a concentration of 40 μg/μL was added. The particle solution was lyophilized in the presence of sucrose at a 5% (w/v) ratio to prevent particle aggregation that could compromise resuspension.

### 2.2. Characterization of the Nanoparticles

#### 2.2.1. Determination of the Size and Surface Charge of the Nanoparticles

The nanoparticle size was examined via dynamic light scattering, and the surface charge was analyzed via electrophoretic particle movement (Zetasizer ZS90, Malvern). Measurements were performed with 1:1000 dilutions of particles in Milli-Q water. The refractive and absorption indices (RIs) were 1.330 and 0.001, respectively. For the dispersing medium, a viscosity value of 0.8872 and an RI value of 1.330 were used. The results were analyzed via Zetasizer software version 7.11.

#### 2.2.2. Fourier Transform Infrared Spectroscopy (FTIR)

The interaction between the different reagents in the nanoparticles was examined via 100 μL samples on a Thermo Scientific Nicolet 6700 instrument, with 40 scans and a resolution of 4 cm^−1^. Spectroscopy was performed over a wavelength range of 4000–600 cm^−1^. The data obtained were processed via OMNIC FTIR software.

#### 2.2.3. Scanning Electron Microscopy (SEM)

The morphology of the nanoparticles was determined by SEM. The samples were placed on slides treated with a 1% (v/v) sulfuric acid solution for 24 h. The samples were subsequently incubated overnight at 37°C to allow for evaporation. The slides were then observed under a scanning electron microscope at a magnification of 13.0 kV (Hitachi, SU5000).

#### 2.2.4. Pharmaceutical Release Assay

In the release test, 15 mg of nanoparticles were diluted in 50 μL of methanol and 450 μL of 2% acetic acid in water. This facilitated the breakdown of the nanoparticles and the dissolution of the active ingredients in the methanol-acetic acid mixture. The solution containing the active ingredients was then filtered through a 0.22 μm filter and subsequently analyzed using the separation system. The released curcumin and gemcitabine were measured by reverse-phase chromatography on a C18 column at wavelengths of 422 and 275 nm (Agilent 1220 Infinity LC System). Elution was performed via a gradient of 0%–100% acetonitrile for 3 min, followed by 70%–30% acetonitrile for 9 min, at a flow rate of 0.3 mL/min.

### 2.3. Determination of the Cytotoxic Potential of the Nanoparticles

The cell lines (A549 and B16F10) were obtained from the American Type Culture Collection (ATCC, Manassas, VA, USA) and maintained in Dulbecco's modified Eagle's medium (Sigma–Aldrich, D5523) supplemented with 10% fetal bovine serum (Gibco, F2442-100) and 1% antibiotic–antimycotic mixture (containing 10,000 units of penicillin, 10 mg of streptomycin, and 25 μg of amphotericin per mL; Sigma–Aldrich, A5955). The A549 cells were used as a model of non-small-cell lung adenocarcinoma, while the B16F10 cells were selected for their potential to form lung metastases.

#### 2.3.1. MTT Assay

A549 cells were seeded in a 96-well plate at 5 × 10^3^ cells per well, and B16F10 cells were seeded at 3 × 10^3^ cells per well, suspended in 100 μL of DMEM medium. Curcumin (Sigma–Aldrich, 34,860) was tested at concentrations ranging from 5 to 40 μM, and gemcitabine (AccogemMR, Accord) was tested at concentrations from 0.05 to 1 μM, both diluted in the culture medium supplemented with 5% FBS and 0.6% methanol. Treatments were incubated for 48 h, and then all were treated with 20 μL of MTT solution (5 mg/mL; Sigma–Aldrich, M2128) and incubated for an additional hour. Afterward, the MTT was discarded by decanting the plates, and 100 μL of DMSO were added to all wells. After homogenization, the samples were analyzed at 570 nm using a Benchmark Plus Bio-Rad microplate reader. The half-maximal inhibitory concentrations (IC_50_) for curcumin and gemcitabine were determined based on data obtained from the MTT assay. Absorbance values were analyzed by fitting a nonlinear dose-response sigmoidal curve using the four-parameter logistic (4PL) model in GraphPad Prism 8.

#### 2.3.2. Pharmacology Interaction

Treatments were performed using all possible combinations of curcumin (5 and 10 μM) and gemcitabine (0.05 and 0.1 μM). Each combination was tested in triplicate following a 48-h incubation period. Drug interaction analysis was conducted using CompuSyn software. A combination index (CI) value below 0.9 was interpreted as synergistic, values between 0.9 and 1.1 were considered additive, and values greater than 1.1 indicated antagonism.

#### 2.3.3. Viability Analysis of Peripheral Blood Mononuclear Cells (PBMCs)

PBMCs were obtained by venipuncture from a healthy donor and collected in an EDTA tube. The PBMCs were isolated using the Histopaque protocol. PBMCs were cultured in a 96-well plate at a density of 4 × 10^4^ cells per well and suspended in 100 μL of the RPMI medium (Sigma–Aldrich, R8758) supplemented with 10% fetal bovine serum and 1% antibiotic–antimycotic mixture. The drug concentrations used were 5–40 μM for curcumin and 0.05–1 μM for gemcitabine. The samples were incubated for 48 h. Subsequently, 10 μL of resazurin solution (Invitrogen, R12204) was added to each well, and the plates were incubated for an additional 4 h. The samples were analyzed using a fluorometer (Thermo Fisher Varioskan LUX) with an excitation wavelength of 560 nm and an emission wavelength of 590 nm.

#### 2.3.4. Staining With Acridine Orange and Ethidium Bromide (AO/EB)

The cells were cultured on sterile slides and treated with curcumin (20 μM), gemcitabine (0.2 μM), or a combination of both (20 μM curcumin and 0.2 μM gemcitabine) for 48 h. The medium was then removed, and the coverslips were washed with PBS. Subsequently, the samples were treated with 25 μL of a solution of AO (100 μg/mL) and EB (100 μg/mL) diluted in PBS. The samples were analyzed under a fluorescence microscope (Carl Zeiss Axio Observer, LLC).

#### 2.3.5. Analysis of Cell Death by Flow Cytometry

The cells were seeded at 2 × 10^4^ cells per well in a 24-well plate and incubated for 24 h. The cells were then treated with 20 μM curcumin, 0.2 μM gemcitabine, or their combination and incubated for an additional 24 h. The cells were stained with a mixture of Annexin V-FITC (Ann-V; 2.5 μL) and propidium iodide (PI; 5 μL) in 200 μL of 1X PBS at pH 7.4. The samples were incubated for 15 min at 4°C in the dark and analyzed via a flow cytometer (BD Accuri C6).

## 3. Results

### 3.1. Nanoparticle Formulation

**Figure 1:**
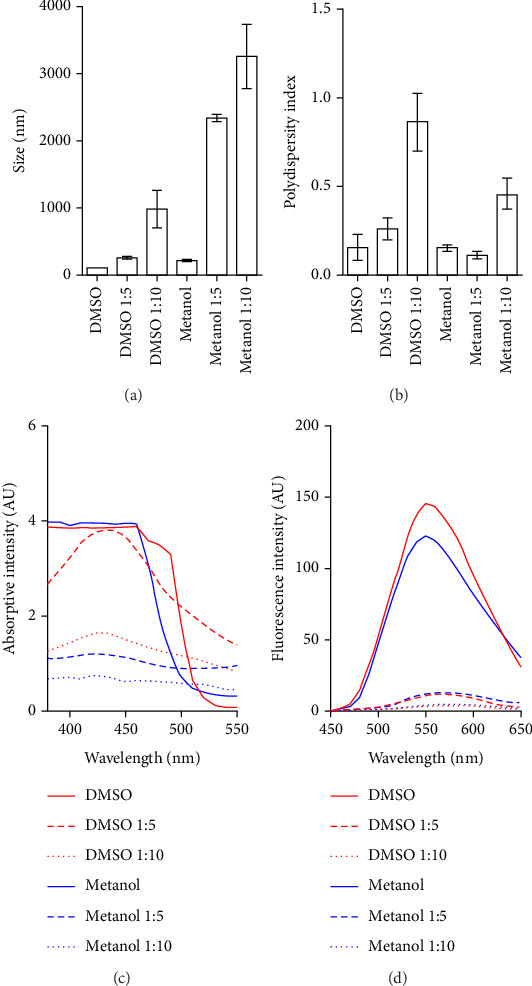
Particle size and property analysis. (a) Particle size, (b) polydispersity index, (c) spectroscopic properties, and (d) fluorescence of different curcumin solutions in DMSO and methanol, as well as their proportions in 0.1 N acetic acid.

Curcumin is difficult to integrate into the ionic gelation system due to its hydrophobic nature, necessitating a method to improve its dispersion. Methanol and DMSO were used as solvents to maintain a stable suspension of curcumin. In 2.5 mg/mL solutions, curcumin sedimented at 2500 and 5000 rpm but remained uniformly suspended at 1000 rpm. Particle size analysis revealed that curcumin diluted in DMSO and its mixtures with acetic acid had smaller particle sizes compared to curcumin diluted in methanol and its mixtures with acetic acid (Figure 1(a)). However, methanol produced more homogeneous particle sizes with lower polydispersity indices than DMSO (Figure 1(b)). In spectroscopic and autofluorescence evaluations, curcumin–DMSO mixtures and their dilutions showed higher absorbance and fluorescence values than the curcumin–methanol mixtures (Figures 1(c), 1(d)). Finally, the use of DMSO did not yield reproducible results during ionic gelation, and particle sizes exceeded 7 microns (Np-DMSO), with negative zeta potential values and a polydispersity index close to one. For all parameters measured in particles synthesized with DMSO, the standard deviations were significantly high, in some cases exceeding the mean value (Table 1). As a result, methanol was chosen as the dispersing agent for curcumin.

### 3.2. Design and Characterization of the Nanoparticles

The physicochemical properties of chitosan (Np-Q), chitosan with curcumin (Np-Cur), and chitosan with gemcitabine (Np-Gem) particles were analyzed after synthesis and lyophilization. The size of the synthesized particles ranged from 180.80 to 188.86 nm, while the size of the lyophilized particles with sucrose (Np-Q + S, Np-Cur + S, and Np-Gem + S) ranged from 181.2 to 197.83 nm. Only Np-Cur and Np-Gem showed significant size differences between the two processes (*p*=0.001). The surface charge of the particles ranged from 15.7 to 16.33 mV, while the surface charge of the lyophilized particles ranged from 11.83 to 13.1 mV. Sucrose significantly reduced the surface charge in all three cases (*p*=0.001 and *p*=0.0001). The polydispersity index was below 0.3 in all cases (Table 1).

**Figure 2:**
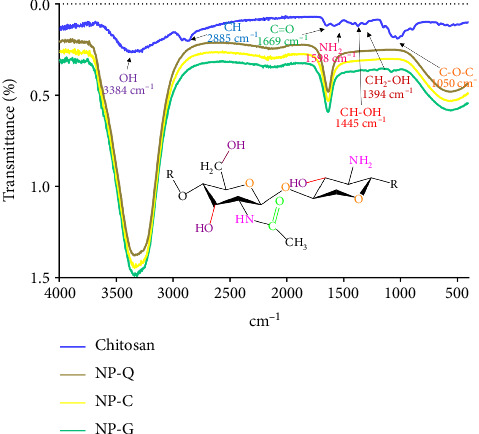
Spectra obtained for commercial chitosan, Np-Q, Np-Cur, and Np-Gem, with identification of the functional groups present.

**Figure 3:**
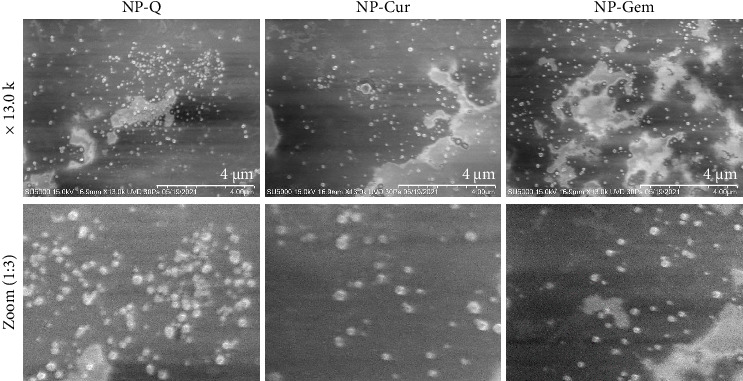
Determination of the morphology of Np-Q, Np-Cur, and Np-Gem by scanning electron microscopy. Images were taken at × 13,000 magnification.

To confirm the chitosan–TPP interaction in ionotropic gelation, an FTIR test was performed. The analysis of the spectra for the three particles (Np-Q, Np-Cur, and Np-Gem) showed that they were identical. A deformation in the intensity of the amino group at 1650 cm^−1^ was observed. Ionic gelation was evident from the deformation of the amino group in the 1550–1600 cm^−1^ region (Table 2 and Figure 2), which shifted to the left and increased in intensity (Figure 2). Additionally, an increase in intensity was observed between 3500 and 3000 cm^−1^. SEM revealed that the nanoparticles exhibited an ovoid morphology (Figure 3).

**Figure 4:**
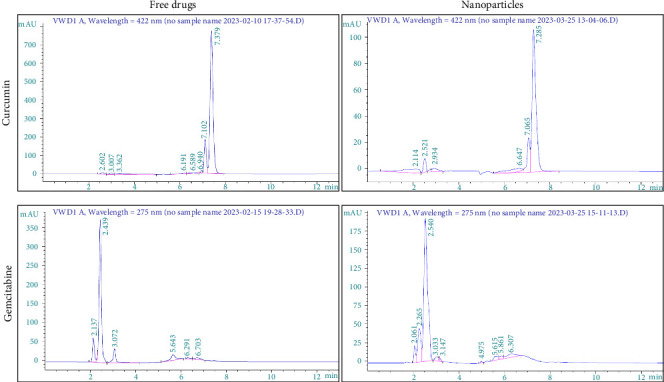
Determination of the presence of curcumin and gemcitabine in the chitosan nanoparticles. Curcumin was identified at a retention time of 7.40 ± 0.4 min, and gemcitabine was identified at a retention time of 2.38 ± 0.4 min.

The quantification of curcumin and gemcitabine using reverse-phase chromatography was based on their respective standard curves, with *R* values of 0.9941 and 0.9822. Curcumin had a retention time of 7.40 ± 0.4 min, while gemcitabine had a retention time of 2.38 ± 0.4 min (Figure 4).

### 3.3. Cytotoxic Effects of Free and Nanoparticulated Curcumin–Gemcitabine

**Figure 5:**
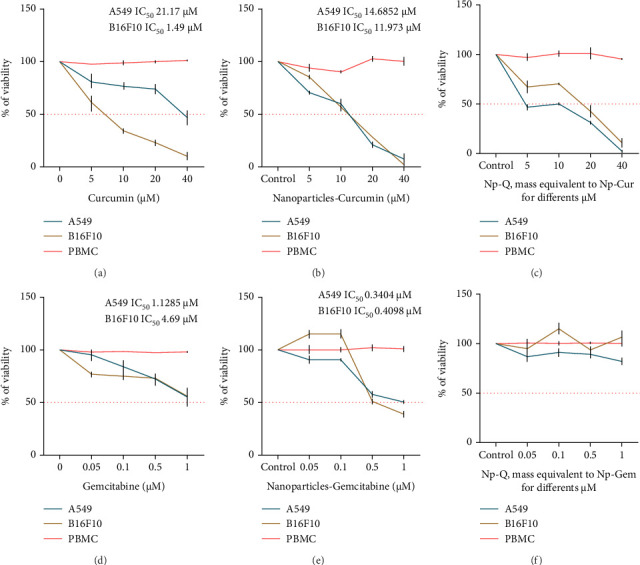
Evaluation of the viability of A549, B16F10, and PBMC cells treated with free curcumin (a) and Np-Cur (c) at concentrations of 5, 10, 20, and 40 μM; free gemcitabine (b) and Np-Gem (d) at concentrations of 0.05, 0.1, 0.5, and 1 μM; and the Np-Q control (e and f). All the treatments were incubated for 48 h.

To evaluate the cytotoxic effects of curcumin and gemcitabine on A549 and B16F10 cell lines, both were treated with varying drug concentrations. Curcumin was more effective in the B16F10 cell line, with an IC_50_ of 8.89 μM (Figure 5(a)), whereas the A549 cell line was more resistant, with an IC_50_ of 21.17 μM. In contrast, gemcitabine induced a dose-dependent decrease in viability, with an IC_50_ of 1.1285 μM in A549 cells and 4.69 μM in B16F10 cells (Figure 5(b)). PBMCs from healthy individuals were used as controls, and no significant cytopathic effects were observed after treatment with curcumin or gemcitabine (Figure 5).

Compared to free curcumin treatment, Np-Cur treatment in B16F10 cells showed an IC_50_ of 11.973 μM, indicating a slight 34.68% decrease in cytotoxic potential (Figures 5(a) and 5(c)). In contrast, the IC_50_ in A549 cells was 14.6852 μM, representing a 30.63% increase in cytotoxic potential compared to the IC_50_ calculated for free curcumin, as shown in Figure 5(c). Compared to cells treated with free drugs, the IC_50_ values of Np-Gem in A549 and B16F10 cells were 0.3404 and 0.4098 μM, respectively, indicating reductions of 69.83% and 91.26% in IC_50_ values (Figure 5(d)). An interesting result was observed at concentrations of 0.05 and 0.1 μM, where Np-Gem induced an increase in cell viability, reaching values of 114.75 ± 3.79% and 115.43 ± 3.85%, respectively.

Finally, Np-Q caused a loss of viability starting from a mass equivalent to 5 μM Np-Cur and continuing up to a mass equivalent to 40 μM. In the case of Np-Q with a mass equivalent to Np-Gem, a slight decrease in viability was observed, but it always remained above 82.12 ± 3.48% in both cell types (Figures 5(e) and 5(f)). PBMCs did not exhibit cytotoxic effects when treated with any of the formulated particles (Figure 5).

The pharmacological interaction analyses of the free drugs revealed a differential response between B16F10 and A549 cell lines when exposed to combinations of curcumin and gemcitabine. In A549 cells, an antagonistic effect was predominantly observed, with CI values exceeding 1.1. Notably, the combination of 10 μM curcumin with 0.05 μM gemcitabine yielded a CI of 4.57, indicating a strong antagonistic interaction. In contrast, the combination of 5 μM curcumin with 0.1 μM gemcitabine showed an additive effect. Conversely, in B16F10 cells, combinations of 5 μM curcumin with both 0.05 and 0.1 μM gemcitabine exhibited synergistic effects. Meanwhile, combinations containing 10 μM curcumin resulted in additive interactions, with CI values ranging from 0.9 to 1.1 (Table 3).

The pharmacological interaction analyses of the nanoencapsulated formulations revealed a predominantly antagonistic profile in both cell lines, with varying degrees of intensity. In A549 cells, all combinations exhibited strong antagonistic effects, with the most pronounced observed for the combination of 5 μM curcumin and 0.1 μM gemcitabine, which showed a CI value of 17.22. In contrast, B16F10 cells also displayed antagonistic effects with the 10 μM curcumin combinations, although these were less intense than those observed in A549. Combinations containing 5 μM curcumin, however, exhibited additive effects, with CI values close to 1 (Table 4).

### 3.4. Morphological Analyses of Cell Death Induced by Curcumin–Gemcitabine Nanoparticulation

**Figure 6:**
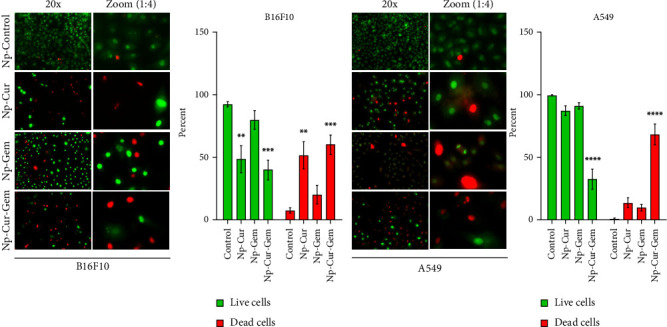
Acridine orange and ethidium bromide staining of cells treated with Np-Q, Np-Cur (20 μM), Np-Gem (0.2 μM), and their combination. Micrographs were taken at 20x magnification after 48 h of incubation with the treatments.

Staining with AO/EB enabled the simultaneous assessment of cell viability, membrane integrity, and nuclear morphology in A549 and B16F10 cell lines following treatment with curcumin-loaded nanoparticles (Np-Cur), gemcitabine-loaded nanoparticles (Np-Gem), and their combination (Np-Cur-Gem). Viable cells exhibited green fluorescence (intact membranes), whereas nonviable cells displayed red fluorescence, indicating loss of plasma membrane integrity. In the B16F10 cell line, the control group exhibited 92.41 ± 2.05% viable cells. Treatment with Np-Cur significantly increased the proportion of nonviable cells to 51.50 ± 10.74%. Np-Gem induced a milder cytotoxic effect, with 20.07 ± 7.52% nonviable cells. As observed in A549 cells, the combined treatment (Np-Cur-Gem) elicited the highest level of cytotoxicity, with 60.09 ± 7.83% nonviable cells. Signs of advanced nuclear damage and extensive membrane disruption were evident. In the A549 cell line, the control group showed a high proportion of viable cells (99.24 ± 0.5%) with typical fusiform morphology. Np-Cur treatment decreased viability to 86.80 ± 4.03%, while Np-Gem induced 9.33 ± 2.52% cell death. In contrast, the combined treatment (Np-Cur-Gem) produced a pronounced cytotoxic effect, resulting in 67.80 ± 8.23% nonviable cells. This group exhibited nuclear condensation, fragmentation, and intense red fluorescence, along with a subset of cells displaying intermediate red/yellow fluorescence, indicative of progressive cellular damage (Figure 6).

### 3.5. Dual Staining-Based Detection of Cell Death via Ann V/PI Flow Cytometry

**Figure 7:**
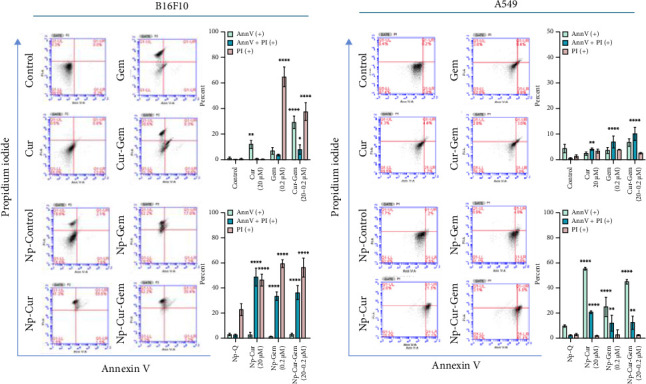
Analysis of cell death by flow cytometry, PI, and Ann-V staining of A549 and B16F10 cells treated with free curcumin and Np-Cur at concentrations of 5, 10, 20, and 40 μM; free gemcitabine and Np-Gem at concentrations of 0.05, 0.1, 0.5, and 1 μM; and the Np-Q control. All the treatments were incubated for 48 h.

To evaluate cell death induced by curcumin and gemcitabine nanoparticles in the cell lines, Ann-V/PI staining was used. Treatment with curcumin or gemcitabine, either in free form or encapsulated in nanoparticles, produced markedly different effects on cell death induction in both cell lines. The B16F10 cell line was more sensitive to treatments than the A549 cell line. Differences were also observed in the staining patterns, with B16F10 cells exhibiting a higher proportion of double-positive cells (Ann-V and PI) following nanoparticle treatment compared to free drugs. In contrast, A549 cells showed a greater proportion of Ann-V only positive cells across the different treatments. Additionally, treatment with both free and encapsulated gemcitabine resulted in a higher percentage of PI only positive cells, as shown in Figure 7.

## 4. Discussion

Curcumin tends to form aggregates in solutions, affecting its optical properties of absorption and fluorescence. The solvent used to dissolve curcumin influences its aggregation and optical properties [30]. Curcumin has a structure similar to other coumarins, such as diphenylhexatriene, which also exhibits this behavior [31, 32]. Due to its predominantly hydrophobic nature, curcumin is expected to dissolve better in less polar solvents. The solvents used in this study were methanol and DMSO, with polarity indexes ranging from 5.1 to 7.2 [33]. Both solvents allowed the suspension of curcumin. Methanol has fewer toxic effects on eukaryotic cells, while DMSO, even at low concentrations, affects gene expression and cellular differentiation and induces epigenetic alterations [34, 35]. Moreover, DMSO hinders the solubility of chitosan processed with acetic acid [36]. Or for these reasons, methanol was selected as the solvent to maintain the suspension of curcumin during synthesis.

The nanoparticles were produced through ionic gelation, and the interaction of the chitosan amino group with TPP was determined. This was evidenced by a shift of the chitosan amino group from the 1585–1590 cm^−1^ region to the 1645–1655 cm^−1^ region, along with an increase in this signal [37]. The amino groups on the Np-Q generate a positive surface charge, which influences their absorption due to better interaction with the negative charge of the plasma membrane caused by phospholipids [38, 39]. Nanoparticles with surface charge values between 2 and 40 mV adhere better than those with lower surface charges [40, 41]. Additionally, phagocytic cells tend to internalize negatively charged particles, while nonphagocytic cells tend to internalize positively charged particles more rapidly [42–44]. Therefore, Z-potential values between 10 and 20 mV ensure that Np-Q, Np-C, and Np-G can be rapidly assimilated by nonphagocytic lung epithelial cells and not eliminated by phagocytic immune system cells.

The optimal size for nanoparticles used in cellular therapies is between 20 and 50 nm, as they can be better absorbed by nonphagocytic cells [45, 46]. However, this size limits the amount of drug that can be incorporated into the nanoparticles. Therefore, nanoparticles between 50 and 150 nm have been studied, as they can enter cells via caveolin- or clathrin-mediated endocytosis [47, 48]. Micrometer-sized particles are more susceptible to phagocytosis [47, 49]. The nanoparticles generated in this study had sizes ranging from 180 to 200 nm, like those reported for other Np-Q obtained by ionic gelation. This size range has also been reported to favor internalization through clathrin-mediated endocytosis, while simultaneously avoiding phagocytosis by immune cells [50, 51]. Additionally, nanoparticles smaller than 200 nm have been identified to show colloidal stability and sustained drug release, an effect observed in Np-Q loaded with doxorubicin or curcumin [52, 53]. The difference in particle size when encapsulating compounds of different polarities can be explained by factors such as hydrophobicity, viscosity of the precursor solution, and agitation conditions during synthesis [54, 55]. The morphology of these nanoparticles was ovoid and has been previously reported for Np-Q used to encapsulate curcumin and gemcitabine [56–60], also coinciding with observations for nanoparticles produced by ionic gelation and subsequent centrifugation, where elliptical or oval shapes are commonly observed as a result of the granulation process [61, 62]. Spherical or oval nanoparticles are internalized more efficiently by nonphagocytic cells via endocytic mechanisms, compared to elongated or irregular-shaped particles, which are preferentially captured by phagocytic cells [63, 64]. Based on the results and the evidence in the literature, the nanoparticles obtained in this study have the potential to be endocytosed in A549 and B16F10 cells.

To understand the effects of the encapsulated compounds, their potential in free form was first evaluated. The solvents used to dilute curcumin influence its cytotoxic effect [65–67], due to the stability it presents in some organic solvents [68]. Therefore, the cytotoxic effect varies depending on whether methanol or DMSO is used. The viability of healthy cells was unaffected by free curcumin or gemcitabine at the concentrations used. Similar results have been reported in previous studies with murine vascular smooth muscle cells, fibroblasts, endothelial cells, and PBMCs, where curcumin was shown to be nontoxic up to concentrations of 40 μM [69–71]. However, several studies have identified that curcumin has cytotoxic properties affecting A549 and B16F10 cells [72–74], with B16F10 cells being more sensitive to the cytotoxic effect of curcumin, with viability below 50% at concentrations as low as 5 μM [72]. These findings are consistent with the results obtained in this study. In contrast, A549 cells were more sensitive to gemcitabine, with an IC_50_ of approximately 1.12 μM, a result similar to that reported in other studies [75]. However, our results differ from those reported by some authors, where the IC_50_ of gemcitabine in A549 cells exceeds 90 μM. This discrepancy could be due to the presence of cells that have developed resistance mechanisms [76–78].

Regarding the cytotoxic efficacy demonstrated in this research, our results are consistent with previous studies where natural compounds, such as marine extracts and other encapsulated phytopharmaceuticals [79, 80], have shown enhanced antiproliferative activity in A549 and B16F10 cell lines. An example of this is the use of lipid nanoparticles loaded with curcumin or marine alkaloids, which have induced significant apoptosis in A549 cells, triggering mechanisms related to oxidative stress and caspase activation [81, 82]. Similarly, Np-Q loaded with doxorubicin or paclitaxel have also shown high efficacy in toxicity presented in lung and murine melanoma cells, reinforcing the usefulness of the drug delivery system used in this study [83, 84]. These comparisons help contextualize the effectiveness of the formulated nanoparticles, highlighting that both the encapsulation system and the physicochemical properties of the encapsulated active ingredients influence the observed biological response. Nanoparticulation of active ingredients improves cellular internalization, enhancing the therapeutic effect by reducing the concentrations of the drug required [85–88]. This same effect was observed in the present study, where the IC_50_ of curcumin was reduced by 34.68% in A549 cells, and the IC_50_ of gemcitabine was reduced between 69.83% and 91.26% in A549 and B16F10 cells when the active ingredients were encapsulated in Np-Q. In contrast, the cytotoxic effect of Np-Cur on B16F10 cells decreased by 30.63%. The increase in cell viability observed at low doses of NP-Gem may be explained by a hormetic response, a phenomenon reported by several chemotherapeutic agents. In such cases, high concentrations have been shown to induce toxicity, whereas low doses can stimulate cell proliferation [89, 90]. Specifically, gemcitabine has been reported to enhance proliferation in lung and pancreatic cancer cells when used at low concentrations [91, 92]. However, the hormetic effect of chemotherapy varies depending on the cell type, tumor microenvironment, drug concentration, and exposure time [89, 92, 93].

The pharmacological interactions between curcumin and gemcitabine showed a strong dependence on both the cell type and concentration. In general, lower concentrations of curcumin favored better pharmacological cooperation, while higher concentrations tended to interfere with gemcitabine's efficacy, likely due to its antioxidant properties [94]. This behavior suggests a hormetic effect that varies depending on the cell line [95–97]. On the other hand, nanoencapsulation drastically altered these interactions, reducing the potential synergy in both cell models. This may be attributed to changes in bioavailability or in the intracellular release profile of the compounds [98]. These findings highlight the importance of considering not only dosage and the cell type but also the pharmaceutical formulation when evaluating therapeutic combinations in cancer.

The concentrations used in the AO/PI assay (0.2 μM gemcitabine and 20 μM curcumin) fall within the range where the MTT assay begins to show a significant decrease in cell viability. However, while the MTT assay indicates a partial reduction in mitochondrial activity, the AO/PI analysis reveals a much higher percentage of cells with irreversible structural damage. This suggests that some of the cytotoxic effects detected by AO/PI may be underestimated by the MTT assay due to the persistence of metabolic activity in cells undergoing cell death [99, 100]. Discrepancies between AO/PI and MTT are expected and reflect the different aspects of cell viability that each technique assesses. Whereas MTT is useful for estimating cellular metabolism, AO/PI provides a more direct view of cellular integrity [101, 102]. The combined use of both techniques, as in this study, allows for a more comprehensive evaluation of the cytotoxic effects of the treatments.

The use of nanoparticle-encapsulated drugs enables the induction of cell death at lower concentrations compared to free drugs, a condition under which necrotic events tend to be less frequent [103, 104]. Although curcumin has been widely reported to induce apoptosis in tumor cells [105, 106], the staining patterns observed in this study—based on Annexin V-FITC/PI and AO/EB—suggest a more complex scenario of cell death mechanisms. In the Annexin V/PI assay, B16F10 cells treated with encapsulated drugs showed a higher proportion of double-positive cells (Annexin V and PI), indicating advanced stages of cell death with loss of membrane integrity. In contrast, A549 cells exhibited a greater percentage of cells positive only for Annexin V across all treatments, which may correspond to earlier stages of regulated cell death, such as apoptosis or necroptosis. Since Annexin V-positive/PI-negative cells still retain membrane integrity and some metabolic activity, they could represent intermediate or even reversible states [100, 107]. Supporting these findings, AO/EB staining revealed consistent patterns, with viable cells emitting green fluorescence and nonviable cells showing red fluorescence due to ethidium bromide uptake [100]. In B16F10 cells, treatment with nanoparticles resulted in a notable increase in red fluorescence and nuclear fragmentation, consistent with irreversible membrane damage and advanced nuclear disintegration [108]. In contrast, A549 cells displayed a higher proportion of yellow or orange fluorescence, suggesting progressive damage with partial membrane compromise [109]. These results align with the idea that B16F10 cells progress more rapidly toward complete membrane rupture, while A549 cells undergo a slower or more modulated form of cell death.

These differences may be attributed to intrinsic characteristics of the cell lines. B16F10 cells are more sensitive to oxidative stress, which could accelerate membrane destabilization [110]. Conversely, A549 cells have been reported to exhibit higher levels of antioxidant enzymes and antiapoptotic proteins [111], potentially delaying the progression of cell death. Moreover, the combination of curcumin and gemcitabine reduced the proportion of cells positive only for Annexin V and increased the proportion of double-positive or PI-only positive cells in both assays, particularly in A549. This may indicate a transition toward more abrupt or less regulated forms of cell death. Given that gemcitabine generates reactive oxygen species (ROS) and curcumin modulates the antioxidant response, including Nrf2 activation [94, 112], their combination could result in antagonistic interactions or trigger alternative responses such as autophagy [97, 113]. Taken together, the combined use of Annexin V/PI and AO/EB reveals that cell death induced by encapsulated agents does not follow a single pathway but instead varies in temporal dynamics and structural progression depending on the cell type. These findings highlight the value of using complementary assays and adopting nuanced interpretations grounded in multiple lines of evidence. From a translational standpoint, understanding these dynamics may contribute to improved pharmaceutical design and support the rational development of synergistic and personalized anticancer strategies.

## 5. Conclusions

This study investigated the effectiveness of encapsulated curcumin and gemcitabine nanoparticles compared to their free drug forms in A549 and B16F10 cells. Analysis of the physicochemical properties and morphology of the nanoparticles revealed that methanol is a more suitable solvent for maintaining curcumin in suspension during synthesis. The formulation and characterization of Np-Q loaded with curcumin and gemcitabine resulted in stable and homogeneous delivery systems with nanometric sizes appropriate for biomedical applications. The nanoparticles exhibited an ovoid morphology, positive surface charges, and a uniform size distribution after lyophilization with sucrose.

The results showed that when curcumin is encapsulated in nanoparticles, its cytotoxic potential is lower than that of the free form in B16F10 cells, whereas it is higher in A549 cells. Moreover, gemcitabine demonstrated a significant increase in effectiveness, with a marked reduction in IC_50_ values in both cell lines. The findings indicate that curcumin and gemcitabine nanoparticles may enhance the cytotoxic response in cancer cells more effectively than the free forms, particularly through mechanisms involving regulated cell death. Overall, the encapsulated nanoparticles enhanced drug delivery and efficacy in tumor cells, suggesting a promising strategy to improve the effectiveness of cancer therapy. This approach may reduce the required drug doses, minimize side effects, and improve the overall safety and efficacy profile in the treatment of certain types of cancer.

Importantly, the pharmacological interaction analysis revealed that drug efficacy is not only influenced by the type of formulation but also by the concentration and the specific cellular context. While synergistic effects were observed in B16F10 cells with free drug combinations at low concentrations, encapsulation altered these interactions, leading predominantly to additive or antagonistic effects. These findings underscore the complexity of drug–drug interactions in nanoparticle-based delivery systems and highlight the need to carefully tailor formulation parameters according to the target cancer type. This study contributes valuable insights into the rational design of combination therapies using nanotechnology for improved selectivity and therapeutic outcomes in oncology.

## Figures and Tables

**Table 1:** Size values, surface charge, and polydispersity indices of Np-Q, Np-Cur, and Np-Gem before and after lyophilization in the presence of sucrose.

Nanoparticles	Size (nm)	Ζ potential (mV)	PDI
Np-DMSO	7847 ± 8878	−9.31 ± 14.45	0.805 ± 0.785
Np-Q	180.89 ± 6.4	15.7 ± 0.48	0.240 ± 0.035
Np-Cur	180.8 ± 6.6	16.33 ± 1.10	0.225 ± 0.033
Np-Gem	188.56 ± 9.33	16.26 ± 0.61	0.220 ± 0.019
Np-Q + S	197.83 ± 2.99	11.83 ± 0.32	0.218 ± 0.003
Np-Cur + S	181.2 ± 1.34	12.43 ± 0.60	0.264 ± 0.008
Np-Gem + S	197.3 ± 2.92	13.1 ± 0.36	0.215 ± 0.020

**Table 2:** Characteristic intensities of the functional groups of chitosan presented in the mid-infrared spectrum.

Intensities (cm^−1^)	Functional groups
3384	OH (hydroxyl)
2938	C-H (Alkyls)
1669	C=O (ester)
1598	NH_2_ (amino group)
1445	CH-OH
1394	CH_2_-OH
1050	C-O-C (ether)

**Table 3:** Therapeutic interaction profile between curcumin and gemcitabine in murine melanoma (B16F10) and human lung (A549) cancer cells.

Treatments	A549		B16F10	
	CI	Interpretation	CI	Interpretation
Cur + Gem (5–0.05 μM)	2.20488	Antagonism	0.55550	Synergism
Cur + Gem (5–0.1 μM)	1.13272	Additive	0.58804	Synergism
Cur + Gem (10–0.05 μM)	4.57571	Antagonism	1.04992	Additive
Cur + Gem (10–0.1 μM)	1.72771	Antagonism	1.06454	Additive

Abbreviation: CI, combination index.

**Table 4:** Therapeutic interaction profile of curcumin- and gemcitabine-loaded nanoparticles in murine melanoma (B16F10) and human lung (A549) cancer cells.

Treatments NP	A549		B16F10	
	CI	Interpretation	CI	Interpretation
Cur + Gem (5–0.05 μM)	5.50028	Antagonism	0.98355	Additive
Cur + Gem (5–0.1 μM)	17.2274	Antagonism	1.07224	Additive
Cur + Gem (10–0.05 μM)	1.89723	Antagonism	1.62405	Antagonism
Cur + Gem (10–0.1 μM)	4.84362	Antagonism	2.04832	Antagonism

Abbreviation: CI, combination index.

## Data Availability

The data that support the findings of this study are available from the corresponding author upon reasonable request. The data are not publicly available due to privacy or ethical restrictions.
